# Oncogenic Signaling Induced by HCV Infection

**DOI:** 10.3390/v10100538

**Published:** 2018-10-02

**Authors:** Alessia Virzì, Armando Andres Roca Suarez, Thomas F. Baumert, Joachim Lupberger

**Affiliations:** 1 Inserm, U1110, Institut de Recherche sur les Maladies Virales et Hépatiques, 67000 Strasbourg, France; virzi@unistra.fr (A.V.); andres.roca-suarez@etu.unistra.fr (A.A.R.S.); thomas.baumert@unistra.fr (T.F.B.); 2 Université de Strasbourg, 67000 Strasbourg, France; 3 Pôle Hépato-digestif, Institut Hospitalo-universitaire, Hôpitaux Universitaires de Strasbourg, 67000 Strasbourg, France

**Keywords:** signaling, cancer, HCV, HCC, chemoprevention, liver disease

## Abstract

The liver is frequently exposed to toxins, metabolites, and oxidative stress, which can challenge organ function and genomic stability. Liver regeneration is therefore a highly regulated process involving several sequential signaling events. It is thus not surprising that individual oncogenic mutations in hepatocytes do not necessarily lead to cancer and that the genetic profiles of hepatocellular carcinomas (HCCs) are highly heterogeneous. Long-term infection with hepatitis C virus (HCV) creates an oncogenic environment by a combination of viral protein expression, persistent liver inflammation, oxidative stress, and chronically deregulated signaling events that cumulate as a tipping point for genetic stability. Although novel direct-acting antivirals (DAA)-based treatments efficiently eradicate HCV, the associated HCC risk cannot be fully eliminated by viral cure in patients with advanced liver disease. This suggests that HCV may persistently deregulate signaling pathways beyond viral cure and thereby continue to perturb cancer-relevant gene function. In this review, we summarize the current knowledge about oncogenic signaling pathways derailed by chronic HCV infection. This will not only help to understand the mechanisms of hepatocarcinogenesis but will also highlight potential chemopreventive strategies to help patients with a high-risk profile of developing HCC.

## 1. Introduction

Tumor-inducing viruses represent a considerable field of study for the comprehension of molecular carcinogenesis. Several oncogenes were first discovered in association with retroviruses and then associated with most forms of cancer [1,2]. The study of virus-coded oncogenes also led to the discovery of canonical signaling pathways and the understanding of elementary cellular processes. Several viruses are considered as oncogenic viruses as they are associated with human cancer, e.g., human papilloma virus (HPV), Epstein–Barr virus (EBV), human herpes virus 8 (HHV8), Merkel cell polyomavirus (MCPyV), human T-lymphotropic virus (HTLV-1), hepatitis B virus (HBV), and hepatitis C virus (HCV) [3].

Infection with oncogenic viruses generally leads to the disruption of genetic and epigenetic homeostasis and DNA repair mechanisms. In addition, some viruses stimulate the proliferation of cancer stem cells (CSCs), which are involved in cancer initiation, progression, and chemotherapy resistance [3]. Oncogenic viruses have a direct and indirect impact on carcinogenesis [4]. At least four HCV proteins (core, NS3, NS5A, and NS5B) seem to deregulate potentially oncogenic signaling pathways [5]. At the same time, it is beyond question that HCV creates a procarcinogenic environment in the liver by inducing a chronic inflammatory state [6]. In addition, liver disease progression can be favored by several cofactors, including alcohol consumption and coinfection with other viruses such as HBV and human immunodeficiency virus (HIV) [7]. Moreover, HCV infection is implicated in extrahepatic cancers, including B-cell non-Hodgkin lymphomas (NHL) [8] and cancers of the oral cavity, oropharynx, intrahepatic bile duct, pancreas, and kidney [9,10,11,12,13,14,15]. Although the molecular links between HCV and extrahepatic cancers are not well understood, it has been suggested that some of the possible mechanisms behind this association could be related to a chronic immune stimulation in the presence of HCV or to the infection of extrahepatic cell types [16].

**Figure 1:**
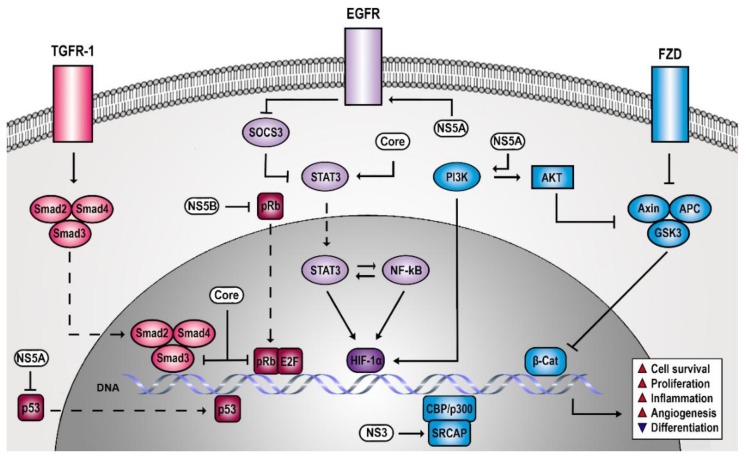
Hepatitis C Virus (HCV)-induced oncogenic signaling. HCV infection creates a procarcinogenic effect through the simultaneous dysregulation of cell survival, proliferation, inflammatory, angiogenic, and differentiation signaling pathways. The tight control of target genes involved in transcriptional regulation and cell cycle progression is altered by HCV via different strategies. Forcing p53 in the cytoplasm, NS5A prevents the gene expression of cyclin-dependent kinase inhibitor p21 (not shown). This cytoplasmic-retention strategy is also shared by NS5B, which traps pRb in the cytoplasm. Consequently, E2F is free to act as transcriptional activator for cell proliferation target genes. Core protein, which is preferentially localized in the cytoplasm, translocates to the nucleus, where it interferes with transforming growth factor-beta (TGF-β) signaling via Smad3 interaction. Epidermal growth factor receptor (EGFR) and mitogen-activated protein kinase (MAPK) signaling are not only required for HCV entry but also represent oncogenic targets for HCV-encoded proteins. Both NS5A and core protein induce the activation of signal transducer and activator of transcription 3 (STAT3) by indirect (inhibiting the suppressor of cytokine signaling 3, SOCS3) and direct mechanisms, respectively. Following its translocation to the nucleus, STAT3 strongly promotes a proinflammatory environment in cooperation with nuclear factor kappa-light-chain-enhancer of activated B cells (NF-κB) signaling. Furthermore, STAT3 and NF-κB, together with PI3K, induce hypoxia-inducible factor 1-alpha (HIF-1α) stabilization, which mediates the transcription of several proangiogenic factors (e.g., vascular endothelial growth factor, VEGF). HCV impairs cell differentiation programs by manipulating Wnt and Notch signaling pathways. NS5A induces a sustained Wnt signaling activation through the PI3K/Akt axis. This leads to the inactivation of a downstream degradation complex and the consequent accumulation of β-catenin in the nucleus, where it activates the expression of cell proliferation-related genes. NS3 stimulates downstream components of Notch pathway by the recruitment of CREB-binding protein (CBP)/p300 complex on Snf2-related CBP activator (SRCAP), repressing cell differentiation programs. TGFR-1: TGF-β receptor 1; FZD: Wnt receptor (Frizzled).

The study of the HCV life cycle revealed several host dependencies of the virus that involve signaling molecules [17,18,19,20,21]. However, it soon became evident that HCV not only requires signaling processes but also actively manipulates host signal transduction with considerable impact on liver pathogenesis. Numerous studies have described signaling cascades that are altered by chronic HCV infection and are potentially involved in carcinogenesis (Figure 1). In the present review, we classify these pathways in three cancer-relevant categories according to their role in cell proliferation/survival, differentiation/adhesion/angiogenesis, inflammatory response, and dissect potential clinical strategies for hepatocellular carcinomas (HCC) chemoprevention and therapy.

## 2. HCV Creates a Persistent Proliferative and Anti-Apoptotic Signaling Environment

Proliferative signaling pathways of mammalian cells are modulated by extracellular factors that engage precise programs of gene transcription and protein regulation [22,23]. Contact inhibition, controlled availability of growth factors, and other physiological feedback systems ensure a tight regulation of the proliferative signaling pathways. Excessive cell proliferation is the key feature of most types of cancers [24]. In general, growth factor and cytokine signaling pathways essentially induce all the primary steps of tumor progression, which include clonal expansion, invasion, angiogenesis, and metastatic formation [25]. Tumor suppressors, such as the cellular tumor antigen p53 and the retinoblastoma-associated protein (pRb), regulate cell proliferation, and their perturbation promotes a persistent activation of the cell cycle machinery [24]. Although HCC proliferative index is generally low, which is one of the reasons why most cytostatics are considered inefficient, there is a clear correlation of HCC risk and proliferative signals in a pretumor state [26].

### 2.1. HCV-Induced Receptor Tyrosine Kinase Signaling Contributes to Liver Cancer Risk

Growth factors like epidermal growth factor (EGF), fibroblast growth factor (FGF), hepatocyte growth factor (HGF), and insulin growth factor (IGF) trigger downstream signal transduction by binding to their specific receptor tyrosine kinase receptors [27]. The cascade of events that follow epidermal growth factor receptor (EGFR) is one of the most widely studied signal transduction pathways [28,29,30]. ErbB-1 and three additional homologous members of the EGFR family (ErbB-2, ErbB-3, ErbB-4), regulate cell proliferation, differentiation, and migration under normal physiological conditions [29]. EGFR itself is critical in epithelial development, and other members of the family have a crucial role in cardiac, mammary glands, and nervous system development and disorders [28,31,32,33]. The EGFR signaling pathway plays a central role also in embryonic development and in the regeneration of stem cells in skin, liver, and gut [34,35]. Moreover, the EGFR signaling pathway is in the spotlight as a driver of cancer risk and progression [26,36,37].

Viruses have developed sophisticated strategies to manipulate EGFR functions (i.e., perturbing EGFR expression, activity, or recycling) [38]. EGFR is a host factor for HCV entry into hepatocytes by regulating the assembly of the coreceptor complex [17,21], viral internalization [39], and membrane fusion [17]. Furthermore, EGFR signaling pathway tempers the antiviral activity of interferon-alpha (IFN-α) by maintaining phosphorylation of signal transducer and activator of transcription 3 (STAT3) through the suppression of a negative feedback regulator (i.e., suppressor of cytokine signaling 3, SOCS3) [40]. It is evident that HCV has a vital interest in maintaining EGFR signaling. Indeed, HCV not only requires EGFR signaling but also actively induces the activation of this pathway during HCV binding and infection [41,42] and prolongs EGFR signaling by perturbing EGFR degradation via NS5A, as reported upon its ectopic expression [43]. This leads to an increased HCC risk in infected patients as persistent EGF signaling is a key driver of liver disease [26].

### 2.2. HCV Increases Cell Survival by Cytoplasmic Retention of p53 and pRb

Proliferative signals seem beneficial for HCV to avoid stress-induced growth arrest and apoptosis, both of which would oppose viral replication and survival [44,45]. The tumor suppressors pRb and p53 regulate cell growth control via their action on cell cycle checkpoints and apoptosis programs [22]. Therefore, pRb, p107, and p130 proteins cooperate with various proteins, including transcription factors of the E2F family required for cellular DNA replication [46,47,48]. The downstream interaction between pRb and E2F causes the inhibition of gene expression by the recruitment of histone deacetylases (HDACs) [49] and other chromatin remodeling factors [50,51,52]. pRb constitutively inhibits the transcriptional activity of E2Fs, whereas it is deactivated after phosphorylation by cyclin-dependent kinases (CDKs). G1 phase CDKs phosphorylate pRb family proteins, which leads to the activation of genes required for S phase entry (i.e., *cyclin E*) [22,24]. In contrast, p53 maintains genetic integrity of cells by blocking cell proliferation in response to stress and DNA damage by activating cyclin-dependent kinase inhibitors (CKIs) [24]. Therefore, p53 accumulates in the nucleus, where it acts as a transcription factor for cyclin-dependent kinase inhibitor 1 (*CDKN1A*) that codes for p21 [53,54]. Thus, it is not surprising that deregulation of p53 function or signaling is associated with many cancers [24]. For example, pRb is a target of viral oncoproteins encoded by adenovirus [55] and HPV [56]. In addition, HCV has developed strategies to suppress pRb [57,58,59]. During HCV infection, NS5B protein retains pRb in the cytoplasm of the hepatocyte, leading to its proteasomal degradation via E6-associated protein (E6AP) recruitment and polyubiquitination [57,59]. The isolated expression of HCV core protein impairs pRb expression in immortalized rat embryo fibroblasts and thereby promotes a E2F-1 activity with impact on cell proliferation and apoptosis [60]. The frequency and the geographic distribution of *TP53* (p53) mutations presumably depend on the variability of aetiological and host susceptibility factors [61,62]. HCV and other viruses have sophisticated strategies to modulate or inhibit p53 signaling [63]. HCV core proteins, NS5A, and NS3 associate with p53 and repress its function without initiating its degradation. HCV core protein, however, seems to act as both activator and a repressor of p53 pathway [64,65,66]. This dual role of core protein may reflect a dose-dependent impact on p53 signaling, depending on the infection model used [67]. In vitro data suggest that the effect of NS3 protein on p53 depends on the HCV genotype [68,69]. Like pRb, virus-induced perturbation of p53 function involves a forced retention in the cytoplasm, which prevents DNA binding of p53. HCV NS5A colocalizes with p53 in the cytoplasmic perinuclear region and sufficiently reduces nuclear p53 concentration to suppress apoptosis. In addition, NS5A expression enforces p53 inhibition via binding to hTAFII32, which is an essential p53 coactivator [70]. In a more indirect manner, HCV proteins perturb the function of essential cofactors of p53 transcriptional activity. Core interacts with DEAD-Box Helicase 3 X-Linked (DDX3X), as observed in an isolated core-expression context [71,72,73]. DDX3X is a target of p53 [74] and modulates *CDKN1A* promoter activity. Furthermore, NS5B binds and relocalizes p53 coactivator DEAD-Box Helicase 5 (DDX5) to the cytoplasm [75,76,77]. However, the findings on p53 signaling during HCV infection have to be interpreted with caution as many of the immortalized cell lines used to study HCV present defects in p53 signaling [6]. For example, Huh7-derived cell lines, which are commonly used due to their high permissiveness towards HCV, accumulate a functionally damaged p53 mutant in the nucleus [78].

### 2.3. HCV Impairs TGF-β Signaling Promoting Epithelial Mesenchymal Transition (EMT)

Cytokines of the transforming growth factor β (TGF-β) superfamily are dimers with conserved structures and exert pleiotropic effects [79]. In physiological conditions, TGF-β acts as a potent growth inhibitor for several types of cells [80,81,82,83,84] and promotes apoptosis in epithelial cells [85]. Consequently, impaired TGF-β may result in cellular hyperproliferation and cancer [86]. In addition, these cytokines stimulate the expression of extracellular matrix components, which promote in vivo fibrosis in different tissues [85,87]. In the liver, TGF-β seems to contribute to all stages of disease development, from early injury through inflammation, fibrosis towards cirrhosis and HCC [88,89]. TGF-β presumably acts as tumor suppressor during the early stage of cancer development but promotes tumor progression, migration, and invasion in advanced HCCs once the tumor cells have acquired resistance to its suppressive proprieties [89,90,91]. Members of the TGF-β superfamily interact with two different receptor types, called type I and type II receptors, which are both required for cellular signaling [85,92,93]. TGF-β binds directly to receptor II, which is constitutively active. This event induces the recruitment of receptor I into the complex that subsequently becomes phosphorylated by receptor II and activate downstream signals [92], which includes SMAD proteins [94,95]. Particularly, the activated type I receptor phosphorylates the intracellular substrate R-SMAD (Smad 2/3 or Smad 1/5/8) that crosses the nuclear membrane after binding co-SMAD (Smad4) [85,89]. Smad4 is a critical effector of intracellular signaling and, like TGF-β, has a dual role as tumor suppressor and promoter of HCC [96]. Once in the nucleus, the SMAD complex regulates the transcription of TGF-β-induced target genes together with essential transcriptional cofactors. The SMAD complex induces a specific gene signature by the canonical TGF-β signaling pathway [97], which provokes growth arrest and proapoptotic signals in an early stage. Later, proliferative and antiapoptotic responses gain the upper hand by crosstalk with growth signaling. This noncanonical TGF-β pathway includes modulation of EGFR, mitogen-activated protein kinase (MAPK), phosphoinositide 3 kinase (PI3K)/Akt, Ras, and Rho-like small GTPases signaling pathways [98,99]. TGF-β can induce epithelial to mesenchymal transition (EMT) in human primary hepatocytes, a program that promotes cell invasion and metastasis [100]. During EMT, the epithelial cells lose their phenotypic features and gain invasive properties to become mesenchymal cells. Physiologically, EMT is indispensable in the context of embryonic development. However, there is increasing evidence that it also plays a role in pathological conditions, probably contributing to metastatic carcinoma development as well [101].

HCV has developed strategies targeting TGF-β signaling, presumably to maintain a proliferative antiapoptotic signaling environment that stimulate the HCV life cycle and prevent stress-induced cell death. HCV infection induces unfolded protein response (UPR), which upregulates TGF-β expression via nuclear factor kappa-light-chain-enhancer of activated B cells (NF-κB) [102,103]. Mainly, the HCV core protein seems to modulate TGF-β signaling (i.e., via an interaction with Smad3) [104,105]. However, HCV does not affect the nuclear translocation of the Smad3/4 complex, suggesting a transient nuclear localization of HCV core protein [105]. An interesting hypothesis suggests that chronic infection provokes the selection of protumorigenic HCV variants in the liver, which strongly interfere with TGF-β signaling. This is supported by the isolation of HCV core variants from HCCs that better resist TGF-β-mediated antiproliferative effects and more intensely promote cell transformation compared to HCV core variants isolated from tissue adjacent to the tumor [105]. Beside its association with SMAD, HCV core expression induces endoglin (CD105) expression on the surface of infected hepatocytes. As a component of the TGF-β receptor complex, endoglin abundance stimulates fibrogenesis and promotes tumor growth and metastasis [106]. Endoglin induces inhibitor of DNA binding 1 (ID1) function via stimulation of ALK-1/SMAD1/5 signaling, which acts as proliferative and antiapoptotic and is a central regulator of CSC development [107]. HCV infection or ectopic expression of viral core enhances the expression of ID1-related markers for survival, proliferation, and CSCs (i.e., BCL2, CyclinD1, HES1, NOTCH1, NANOG, and SOX2 proteins) [106]. Furthermore, endoglin is an angiogenesis marker in patients with HCC [108,109].

## 3. HCV Manipulates Signaling Circuits of Differentiation, Adhesion, and Angiogenesis

A hallmark of HCC development is the dedifferentiation of hepatocytes, which is accompanied by important changes in intracellular communication and nutrients supply. The identification and understanding of stem cell-like cells in cancers has significantly contributed to the current understanding of tumor formation [110]. Even though CSCs share a few key features of normal tissue stem cells (e.g., unlimited proliferative and differentiation ability), they are potentially able to reproduce many of the elements related to cancer initiation, metastasis, and recurrence after therapy [111,112,113]. For HCC, a rare population of CSCs, called liver cancer stem cells (LCSCs), is abundant in tumor tissues and support self-renewal malignant transformation and resistance to chemotherapy [114]. Several LCSCs markers have been identified that have impact on the signaling circuitry, and some of them have been proposed as therapeutic targets for liver cancer treatment [115].

### 3.1. HCV Infection of Hepatocytes Provokes Stem Cell-Like Characteristics

During HCV infection, the virus predisposes cells towards the acquisition of CSC characteristics by the dysregulation of several signaling pathways [116,117]. Many of the characteristic CSC markers (i.e., CD133, CD90, CD44, and EpCAM) are also modulators of signaling pathways, including MAPK pathway, TGF-β mediated EMT, Wnt signaling, which are required to maintain CSC properties [115,118,119,120,121,122,123,124,125,126,127]. Other CSCs markers, such as doublecortin-like kinase 1 (DCLK1), impact microtubule filaments, polarized polymers of α and β tubulin heterodimers that are essential for cellular transport, cell division, and differentiation. DCLK1 is overexpressed in the liver of patients with HCV-associated HCC, while its level is very low or absent in normal hepatocytes. Interestingly, HCV replication, inflammation, and cirrhosis contribute to DCLK1 accumulation in the perinuclear region of the hepatocytes, where it colocalizes with NS5A and microtubule filaments [117]. This suggests that HCV-induced DCLK1 activity promotes microtubule filament polymerization and stabilization [117,128]. The maintenance of the CSC state is principally driven by reactivation of embryonic differentiation programs. Of these, especially Wnt, Notch, and Hedgehog signaling pathways potentially play a role in HCV-induced carcinogenesis [129,130,131,132].

### 3.2. HCV Causes Wnt Upregulation and β-Catenin Accumulation

Wnt pathway is a crucial component for embryonic development and tissue homeostasis [133]. Activation of the pathway starts when Wnt ligands bind to Frizzled (FZD) receptor, a seven transmembrane protein containing an extracellular cysteine-rich ligand-binding domain. When FZD receptor is activated, it inhibits the degradation of β-catenin. This leads to β-catenin accumulation and translocation to the nucleus, where it activates regulators of cell proliferation [134], such as *WISP-1, c-MYC*, and *CCND1* [135,136,137]. Absence of FZD stimulation causes degradation of cytosolic β-catenin by a complex that consists of Axin, adenomatous polyposis coli protein (APC), and two serine/threonine kinases (GSK3β and CK1). Moreover, β-catenin potentiates the expression of ΔN-p73, a repressor of p53 and Tap73 proteins, conferring antiapoptotic and chemoresistance proprieties to HCC cells [134,138,139,140]. Components of the Wnt signaling are frequently mutated in liver cancer [141], which mostly result in β-catenin stabilization [142]. HCV infection manipulates Wnt signaling in multiple ways via its structural and nonstructural viral proteins. Isolated expression of NS5A has been reported to directly promote Wnt signaling by its interaction with PI3K and subsequent activation of Akt. This induces the phosphorylation and inhibition of glycogen synthase kinase 3β (GSK3β), a key component of β-catenin degradation complex [143]. Furthermore, ectopic expression of HCV core protein induces cell proliferation by forcing the expression of *Wnt-1* and its downstream target gene *WISP2*, which induce Wnt signaling [144].

### 3.3. HCV Enhances Notch Signaling by Coactivating Hes-1 Promoter

Notch signaling suppresses cell differentiation, and it is involved in the maintenance of CSCs [145,146]. Notch ligands and receptors are both EGF-homologous transmembrane proteins mediating intercellular communication, cell proliferation, differentiation, and apoptosis [147]. Its impact on the cell is defined by the cellular microenvironment and its crosstalk with different signaling pathways [148]. To be activated, Notch receptors undergo a sequence of proteolytical cleavage upon interaction to a cell-bound ligand exposed on the surface of neighboring cells. Subsequently, this leads to the release of the Notch intracellular domain (NICD) and its translocation into the nucleus. Nuclear NICD associates with numerous cofactors and repressors, fine-tuning its transcriptional activity [147]. The complex orchestrates transcription of Notch target genes that regulate cell differentiation, such as hairy enhancer of split (*HES1*) [149], HES-related proteins (*HEY*), Notch-regulated ankyrin repeat protein (*NRARP*) [150], cyclin D1 (*CCND1*) [151], *c-MYC* [152,153,154,155], and receptor tyrosine-protein kinase erbB-2 (*ERBB-2*) [156]. In addition, Notch influences inflammation and metabolism by contributing to the activation of NF-κB [157] and peroxisome proliferator-activated receptor (PPAR) [148].

HCV infection interferes with Notch signaling and thereby contributes to hepatocarcinogenesis. Under isolated expression condition, NS3 protein binds to Snf2-related CBP activator protein (SRCAP) and cooperatively enhances Hes-1 promoter activity [158]. This leads to increased Notch-induced *HES1* expression [159], a transcriptional repressor of cell differentiation [160], suggesting that HCV promotes a dedifferentiated CSC-like state of infected hepatocytes.

### 3.4. HCV-Induced Liver Damage Promotes Hedgehog Signaling

The Hedgehog pathway (Hh) is involved in the regulation of several morphogenic key functions, such as proliferation, survival, migration, and differentiation [161]. The Hedgehog ligands are essential during morphogenesis and embryogenesis processes as well as for the maintenance of stem cell homeostasis during adulthood [162]. Importantly, Hh pathway plays an essential role in adult liver repair and regeneration [163] and is implicated in several types of liver cancer, such as gallbladder cancer [164], cholangiocarcinoma [165,166,167], hepatoblastoma [168], and HCC [169]. Probably, the production of Hh ligands is favored by the accumulation of liver damage markers (i.e., platelet-derived growth factor (PDGF), TGF-β, and EGF) [170,171,172].

In patients with viral hepatitis, the Hh pathway is found to be induced [173], which presumably reflects tissue damage and liver regeneration during chronic infection. Interestingly, the permissiveness of cells to HCV replication seems to positively correlate with Hh pathway activity [174], suggesting that liver regeneration and a profibrotic environment may promote HCV infection. This is supported by the identification of additional key regulators of liver regeneration that are activated by HCV infection, including EGFR [17,41,42] and IL-6/STAT3 [175] signaling. Moreover, the presence of Hh activity promotes EMT in crosstalk with TGF-β and Wnt signaling [176], which once more highlights the relevance of EMT induction for HCV and its consequences for HCV-associated liver pathogenesis and HCC development.

### 3.5. HCV Promotes Angiogenesis via VEGF and HIF-1α Stabilization

Angiogenesis is a complex growth factor-dependent process responsible for the formation of new vessels from existing vascular trees [177,178]. Physiological angiogenesis is maintained by the balance between proangiogenic and antiangiogenic factors [179]. In pathological conditions, new growth in the vascular web is relevant as the proliferation of cancer cells and metastasis depend on a satisfactory source of oxygen and nutrients as well as waste removal from organs and tissues [180]. Several angiogenic growth factors are elevated in HCC patients, i.e., vascular endothelial growth factor A (VEGF-A), angiopoietin-2 and PDGF [181,182]. HCV infection leads to the development of hepatic angiogenesis, which significantly contributes to HCC progression and invasion [183]. This proangiogenic state is reversed in the livers of patients after viral clearance [184]. Vascular endothelial growth factor (VEGF) is a key regulator of angiogenesis in both normal and neoplastic tissues. Its expression and function are modulated by cytokines and other factors, such as the hypoxia-inducible factor 1α (HIF-1α) [182,185,186]. HCV infection leads to the stabilization of HIF-1α, mediated via oxidative stress and the induction of hypoxia [187]. In addition, the activation of PI3-K/Akt, Erk1/2, NF-κB, and STAT3 is necessary for hypoxia-inducible factor 1-alpha (HIF-1α) stabilization, which leads to the stimulation of VEGF [187]. HCV core protein triggers hepatic angiogenesis by a mechanism that involves crosstalk of multiple pathways, which is reflected by altered marker expression for hepatic angiogenesis, including TGF-β2, VEGF, and CD34 expression [185].

## 4. HCV Tweaks Signaling of the Inflammatory Response

Inflammation is an essential physiological response to several distressing stimuli, including infection. Inflammation is also tightly linked to the mechanisms of tissue regeneration and cancer. During chronic inflammation, NF-κB and STAT3 are central regulators of liver inflammation and are frequently associated with increased risk of cancer [188,189]. As part of the immune system, NF-κB contributes to the elimination of transformed cells. In support of this, NF-κB activation during the acute inflammatory response is highly associated with cytotoxic immune cell response [190]. The activation of NF-κB is induced by the IκB kinase (IKK) complex, which mediates phosphorylation and proteasomal degradation of IκB. This allows NF-κB dimers to translocate into the nucleus, where they induce an inflammatory and antiapoptotic response [191]. NF-κB is constitutively active in many types of cancer, promoting tumorigenic processes [192,193,194]. This suggests a dual role of NF-κB as a tumor suppressor and a tumor promoter, depending on the duration and intensity of tissue inflammation. NF-κB is a transcription factor and a central regulator of inflammation and cell survival. In quiescent cells, NF-κB is inactive, blocked by a tight association with inhibitor of NF-κB (IκB). NF-κB is further regulated by post-translational modifications (e.g., phosphorylation), which are important for its activation and crosstalk with other signaling pathways [195]. Moreover, NF-κB activity is influenced by dynamic protein–protein interactions, forming a tight network of feedback loops and interconnections [196]. In addition, STAT3 possesses a dual role as tumor suppressor and oncogene. It is not only a pivotal transcription factor in acute inflammation, but it is also a key element of liver regeneration [197] by regulating cell proliferation, survival, angiogenesis, and chemotaxis [198,199]. STAT3 is induced by a variety of different ligands, including interleukin 6 (IL-6), cardiotrophin-1 (CT-1), leukemia inhibitory factor (LIF), EGF, oncostatin M (OSM), IFN-α, and IFN-β [200]. Engagement of these ligands to their receptors leads to a subsequent recruitment of Janus kinases (JAK1, 2 and 3) and tyrosine kinase 2 (TYK2) that phosphorylate STAT3 [92,201,202,203]. Once phosphorylated, STAT3 forms homo- or heterodimers with STAT1 or STAT5 that translocate to the nucleus and bind specific DNA sequences. Without a doubt, STAT3 phosphorylation is necessary for its transcriptional activity. However, unphosphorylated STAT3 also presents biological functions, such as the expression of cell cycle progression genes [204,205]. NF-κB and STAT3 signaling are closely linked. NF-κB-mediated inflammation induces hepatic IL-6 production and STAT3 signaling [206]. Activated STAT3 in cancer cells binds to the NF-κB complex proteins RelA/p65 and the histone acetyltransferase p300 in the nucleus. As a consequence, p300 reversibly acetylates RelA/p65 dimers [207], which cause its nuclear retention [208]. At the same time, NF-κB can also impair oxidative stress, which is an activator of STAT3 [209]. In most HCC tumors, however, STAT3 activity does not coincide with NF-κB activation [210].

### HCV Affects the STAT3/NF-κB Circuitry to Maintain a Pro-Inflammatory State

One of the most important examples of inflammation-associated cancers is HCC succeeding chronic HCV infection [211]. Compared to HBV infection, where viral genome integration accounts for the majority of HCCs, HCV-induced HCC is linked to liver disease progression from nonalcoholic fatty liver disease (NAFLD), chronic inflammation, fibrosis, and cirrhosis. This therefore suggests that HCV-induced signals promote liver fibrosis and disease progression following a similar disease pattern observed for other aetiologies. Indeed, HCV causes hepatic inflammation and induces complex alterations in host signal transduction [212]. These include deregulation of cytokine, metabolic, and oxidative stress pathways [213]. HCV-encoded proteins also cover an important role in initiating and maintaining this chronic inflammatory state. For instance, NS5A upregulates the expression of cyclooxygenase-2 (COX-2) [213], which promotes chronic inflammation by the synthesis of prostaglandins. It is therefore not surprising that HCV manipulates regulatory signaling of the inflammatory response, including NF-κB [189] and STAT3 [214], and thereby increases the risk of HCC development. HCV induces chronic hepatic inflammation that is mediated by elevated NF-κB activity. However, the question is whether this is simply a consequence of the cellular defense against infection by HCV or whether the virus has an interest in maintaining an inflammatory state for its own benefit. Several lines of evidence suggest that HCV indeed gains from tweaking the outcome of the inflammatory response. For example, HCV infection enhances tumor necrosis factor alpha (TNF-α)-induced cell death by suppression of NF-κB activation involving a mechanism dependent on core, NS4B, and NS5B [215]. At the same time, HCV makes use of parts of the NF-κB signaling by activating IKKα which, independent of NF-κB, induces the expression of lipogenic genes that contribute to core-associated lipid droplet formation [20]. The same is true for STAT3, which is a mediator of inflammation and part of the interferon response against viral infection. STAT3 transcriptional activity is elevated upon HCV infection in livers of patients and in cell culture [175] and is associated with poor prognosis in HCCs [189]. STAT3 is activated by HCV-induced oxidative stress via core, NS2, and NS3 proteins [216] and by the innate antiviral immune response in hepatocytes [40]. Additionally, the presence of HCV not only affects the infected hepatocytes but equally affects the liver microenvironment. Exosomes secreted from HCV-infected cells carrying miR-19a induce STAT3 activation in hepatic stellate cells and favor fibrotic gene expression [217]. STAT3 activation in the context of HCV infection has also been linked to the presence of myeloid-derived suppressor cells (MDSCs), a cell type that favors the expansion of T_reg_ lymphocytes and has been associated with an increased tumor burden in HCC patients [218]. The question then arises as to whether the elevated STAT3 signaling is simply a consequence of infection or whether it is beneficial to the virus. Interestingly, HCV core protein also directly associates and activates STAT3 function, which promotes cell transformation [219], suggesting an important role of STAT3 for HCV. Indeed, HCV has a vital interest in maintaining a persistent STAT3 signaling as STAT3 is a cofactor for HCV infection and tempers the antiviral impact of the interferon response [40].

## 5. Clinical Relevance and Perspectives

Chronic HCV infection is a major cause of HCC, the second most deadly cancer worldwide with only very limited treatment options. HCV-related HCC will remain a major health problem for the next decades, despite the recent development of direct-acting antivirals (DAAs) and their deployment in therapy [220]. Especially in patients with advanced liver disease, the HCC risk cannot be fully reversed after viral cure [221]. This is similar to alcohol-induced liver disease, where the HCC risk during abstinence persists for several years [222]. Although the oncogenic mechanism of alcohol and its carcinogenic metabolite acetaldehyde differ from that of viral hepatitis, it has been suggested that, similar to alcohol [223,224], HCV infection may leave an epigenetic footprint in the host genome. An interesting question is whether this also creates persistent alterations in the host signaling network that maintain an oncogenic pressure to the hepatocyte, like an echo from the chronic infection.

Another point worth mentioning is a suggested increase in tumor recurrence rates in HCC patients after DAA-induced sustained virological response and tumor resection [225,226]. However, these results remain controversial as other groups could not confirm this observation [227,228]. Therefore, whether antiviral treatment in HCC patients leads to a long-term survival benefit is currently unknown, and current guidelines suggest a close surveillance and imaging in these patients [229]. The treatment of HCC is particularly challenging for patient cohorts with moderate and severe liver dysfunction (Child–Pugh Class B or C) in term of toxicity and efficacy as the use of sorafenib for the treatment of Child–Pugh B patients has been questioned [230]. Moreover, the HCC proliferative index is low, which is one of the reasons most cytostatics and small molecules are considered inefficient.

By hijacking the host signaling network, HCV generates a proliferative and antiapoptotic environment, which promotes hepatocyte dedifferentiation and EMT. This forms an optimal environment for the virus to persist but with serious consequences to the host. The signaling pathways deregulated by chronic HCV infection resemble the hallmarks of cancer [231,232], suggesting that HCV-induced oncogenic signaling likely contributes to liver disease progression and hepatocarcinogenesis. Targeting signaling components with therapeutic antibodies or clinical kinase inhibitors in cancer therapy is widely established. The current pharmacological therapy for HCC is essentially based on the multikinase inhibitor sorafenib [233], which is able to increase survival rates of selected HCC patients. Other kinase inhibitors clinically tested include linifanib (VEGFR and PDGFR inhibitor) [234] and erlotinib (EGFR inhibitor) [235]; the latter failed in phase 3 of its clinical trial [236]. The identification of therapeutic targets in established HCCs is difficult because genetic alterations in tumors are highly heterogeneous [237]. Nevertheless, such approach holds promise in the framework of a personalized treatment, and targeting derailed signaling pathways in patients at risk of developing HCCs can be part of novel chemopreventive strategies. In support of this, an important proof-of-concept was demonstrated in 2014 by Bryan Fuchs and colleagues as erlotinib-attenuated fibrogenesis and HCC development in a rat model [26]. Other HCV-modulated signaling pathways (i.e., NF-κB and STAT3) offer interesting opportunities to therapeutic intervention, as well as prevention, especially in the pathological context of HCC [189].

However, this requires new and well-tolerated compounds that allow a long-term administration of kinase inhibitors to patients with advanced liver disease. A deeper understanding of the signaling network of HCV infection will also contribute to a better understanding of general signaling events involved in liver disease progression, given the gene expression profiles in patients at risk of HCC seem to be independent of the underlying aetiology [238]. In future, well-established HCV infection models will be instrumental in highlighting additional deregulated and druggable signaling pathways that are associated with HCC risk. This will help to overcome the lack of appropriate study models of HCC development and contribute to the discovery of novel drivers and drug targets of liver disease and HCC development.
